# Source Attribution of Health Benefits from Air Pollution Abatement in Canada and the United States: An Adjoint Sensitivity Analysis

**DOI:** 10.1289/ehp.1205561

**Published:** 2013-02-22

**Authors:** Amanda Joy Pappin, Amir Hakami

**Affiliations:** Department of Civil and Environmental Engineering, Carleton University, Ottawa, Ontario, Canada

**Keywords:** adjoint sensitivity analysis, health benefits, nitrogen dioxide, ozone mortality, source attribution

## Abstract

Background: Decision making regarding air pollution can be better informed if air quality impacts are traced back to individual emission sources. Adjoint or backward sensitivity analysis is a modeling tool that can achieve this goal by allowing for quantification of how emissions from sources in different locations influence human health metrics.

Objectives: We attributed short-term mortality (valuated as an overall “health benefit”) in Canada and the United States to anthropogenic nitrogen oxides (NO_x_) and volatile organic compound (VOC) emissions across North America.

Methods: We integrated epidemiological data derived from Canadian and U.S. time-series studies with the adjoint of an air quality model and also estimated influences of anthropogenic emissions at each location on nationwide health benefits.

Results: We found significant spatiotemporal variability in estimated health benefit influences of NO_x_ and VOC emission reductions on Canada and U.S. mortality. The largest estimated influences on Canada (up to $250,000/day) were from emissions originating in the Quebec City–Windsor Corridor, where population centers are concentrated. Estimated influences on the United States tend to be widespread and more substantial owing to both larger emissions and larger populations. The health benefit influences calculated using 24-hr average ozone (O_3_) concentrations are lower in magnitude than estimates calculated using daily 1-hr maximum O_3_ concentrations.

Conclusions: Source specificity of the adjoint approach provides valuable information for guiding air quality decision making. Adjoint results suggest that the health benefits of reducing NO_x_ and VOC emissions are substantial and highly variable across North America.

Acute and chronic exposure to ambient air pollution has been directly linked with adverse human health effects, resulting in substantial social and economic burdens worldwide. Several time-series and cohort studies conducted over the past few decades have examined the effects of particulate matter (PM) and gas-phase pollutants on short and long-term mortality and morbidity. In application, the results of such studies have been linked with air quality modeling to estimate the global burden of air pollution (Anenberg et al. 2010; Brauer et al. 2012) and the health impacts of intercontinental pollutant transport (Anenberg et al. 2009) and to evaluate control measures (Tagaris et al. 2010; West et al. 2006).

Ozone (O_3_) is one of the major photochemical oxidants in ambient air whose short-term health effects have been widely researched (e.g., Bell et al. 2005; Burnett et al. 1997; Katsouyanni et al. 2009). In a U.S. multicity study, Bell et al. (2004) estimated that a 10-ppb increase in 24-hr average O_3_ concentration was associated with a 0.52% increase in all-cause mortality. Subsequently, Ito et al. (2005) conducted a meta-analysis of single-city time-series studies worldwide and found a slightly lower estimate of 0.39% for a 10-ppb change in 1-hr maximum O_3_ concentration. In a study of 12 major Canadian cities, Burnett et al. (2004) associated a 30.6-ppb change in 2-day moving average O_3_ concentration with a 2.74% change in nonaccidental mortality. More recent cohort studies on the long-term effects of O_3_ (e.g., Jerrett et al. 2009) suggest that chronic O_3_ exposure may have a stronger influence on mortality as well as the potential to afflict substantially larger societal costs.

Unlike O_3_, there is a lack of consensus concerning the association between nitrogen dioxide (NO_2_) and short-term mortality, due in part to a scarcity of epidemiological evidence (e.g., Latza et al. 2009; Stieb et al. 2008). However, in a Canadian study, Burnett et al. (2004) found that a 22.4-ppb increase in 3-day moving average NO_2_ concentrations was associated with a 2.31% increase in nonaccidental mortality. This association was further examined by Brook et al. (2007), who concluded that NO_2_ is the best single indicator of species in the ambient pollution mixture whose human health effects are not yet fully understood.

Quantification of health effects can be extended to emissions as part of benefit–cost analyses with the use of chemical transport models (CTMs) that relate emission rates to ambient concentrations of pollutants (e.g., Anenberg et al. 2010; West et al. 2006). Model-based studies have traditionally used a scenario-based approach that aims to quantify health effects that would result if emissions from all sources were reduced uniformly or based on a prescribed scenario. Such studies are beneficial in assessing the spatiotemporal distribution of health benefits resulting from prescribed changes in model inputs, but cannot feasibly quantify distinctions between health benefits related to emission reductions from sources in different locations and times.

The quantified relationship between CTM-based model outputs and inputs is referred to as sensitivity analysis in the context of this work. Sensitivity information relates changes in emissions coming from sources [e.g., nitrogen oxides (NO_x_) emissions from motor vehicles or a power plant] to concentrations seen at receptors (e.g., health effects related to O_3_ exposure in a city) and thus estimates how much influence a source has on a receptor. These influences can be attributed to changes in emissions for a group of sources altogether (e.g., the transportation sector), as in previous studies, or instead to emissions coming from each source individually, yielding source-specific information. In a benefit–cost analysis framework, it is beneficial to know the marginal influences of emissions from different source locations on health effects. This kind of source-specific information can be achieved using adjoint or backward sensitivity analysis in CTMs. In this approach, influences on receptors are traced back to individual sources at all locations in preceding times (hence the term backward).

Here, we present a proof-of-concept study for integrating health benefit assessment models and epidemiological data with the adjoint of CTMs (the tool used to conduct adjoint sensitivity analysis) by forming a direct linkage between health effects at a national scale and emission sources at each location. We apply our methodology to estimate the response of national short-term mortality (valuated as an overall “health benefit”) in Canada and the United States from short-term exposure to O_3_ (and NO_2_ in Canada) to emission reductions in each location across North America.

## Methods

*Adjoint sensitivity analysis*. Adjoint sensitivity analysis, within the context of this work, refers to the estimation of influences coming from emissions at individual source locations on short-term O_3_ mortality aggregated across all receptors. The difference between the adjoint approach and more conventional methods for sensitivity estimation is one of perspective and lies in the direction in which sensitivity information evolves through the model in time and space. Conventional methods for sensitivity analysis track influences of a source, or a group of sources (e.g., all power plants), forward in time and space to all receptors (e.g., Canada and the United States), and are therefore referred to as “forward methods.” One such approach is the brute-force method in which emission inputs to CTMs are changed in the model to estimate the resultant distribution of concentrations across all receptor locations and times. With this method, it is a prohibitively costly undertaking to estimate influences of individual sources because each source requires its emissions to be perturbed separately. A particular type of brute-force method, known as zero-out sensitivity analysis, requires emissions from a particular source be set to zero on the premise that removing a source will reveal its overall influence. In contrast, adjoint sensitivity analysis is a “backward method” that calculates influences of each source location on a single receptor or an ensemble of receptors. A single adjoint simulation provides sensitivities of a model output with respect to inputs across all locations and times (e.g., how O_3_-related mortality in Canada changes as a result of a change in emissions in any location) without requiring any perturbations to be made to model inputs themselves.

Detailed explanation of adjoint sensitivity analysis in air quality modeling can be found elsewhere (Hakami et al. 2007; Henze et al. 2007; Sandu et al. 2005); here, we provide a descriptive overview. As mentioned above, the adjoint method can provide information about influences of location-specific sources on a function such as nationwide mortality that depends on concentrations across many receptor locations. This concentration-dependent function is commonly called the adjoint cost function. We define the adjoint cost function as the monetary value of mortality (*M*) resulting from short-term exposure to O_3_ (and NO_2_ in Canada). We use epidemiological concentration response functions, population data, and recorded baseline mortality rates to establish a concentration-based adjoint cost function. Our adjoint sensitivity results, therefore, estimate influences from emissions in different source locations and for different species on nationwide mortality metrics.

Linkage between epidemiological models and adjoint calculations is established through the appropriate definition of the adjoint cost function. A change in mortality valuation (Δ*M*) associated with a change in pollutant concentration (Δ*C*) is often given by

Δ*M* = *M*_0_ × *P* × *V_SL_*(1 – *e*^–βΔ^*^C^*), [1]

where *M*_0_ is the baseline nonaccidental mortality rate, *P* is the population, *V_SL_* is the value of statistical life (VSL), and β is the concentration–response factor based on epidemiological models. VSL is the most common mortality valuation metric and is a measure of an individual’s willingness to pay to reduce their probability of death (Alberini et al. 2006). Studies that have quantified the health benefits of air pollution reduction have often concluded that mortality reduction is the largest contributor (Hubbell et al. 2005).

Adjoint sensitivity calculations are driven by the adjoint forcing term (ϕ) in the same fashion that concentrations are driven by emissions in CTMs. By this analogy, adjoint forcing terms can be regarded as “sources of influence” in the same way that emissions are sources for concentrations. The adjoint forcing term is the local, marginal influence of a change in concentration (*C*) on the adjoint cost function (ϕ = ∂*M*∕∂*C* ≈ Δ*M*∕Δ*C*). The linearized form of Equation 1 results in the approximation of the adjoint forcing term:

ϕ = ∂*M*∕∂*C* ≈ Δ*M*∕Δ*C* ≈ *M*_0_ × *P* × *V_SL_* × β. [2]

Because β is often a small value, the resulting error from this linearization is negligible for all practical purposes. As Equation 2 suggests, adjoint forcing terms, acting as the sources of influence, increase with the size of population. If only mortality valuation due to O_3_ exposure is considered, the forcing term applied would only include a concentration response factor for O_3_, but because O_3_ is influenced by other species in various locations through atmospheric chemistry and transport, emissions of other species [e.g., NO_x_ and volatile organic compounds (VOCs)] are linked to O_3_-related mortality in CTMs.

*Health outcome valuation*. Our estimation of mortality valuation for Canada is based on O_3_ and NO_2_ short-term mortality. The required Canadian data for Equation 2 are extracted from the Air Quality Benefits Assessment Tool (AQBAT) developed by Health Canada (Judek et al. 2006), which considers O_3_ (daily 1-hr maximum) and NO_2_ (24-hr average) to have β-values of 8.39 × 10^–4^/ppb and 7.48 × 10^–4^/ppb, respectively, based on (although not identical to) Burnett et al. (2004). We include NO_2_ in our analysis for Canada based on the recommendation of Brook et al. (2007) and because of its inclusion in the Canadian Air Quality Health Index (AQHI) (Stieb et al. 2008).

For the United States, we used results from different epidemiological studies, based in full or part on U.S. time-series data, to examine the importance of choice of metrics based on different averaging periods. Our default U.S. estimations are based on the widely used β-value of 5.2 × 10^–4^/ppb for 24-hr average O_3_ from Bell et al. (2004), but we also consider a β-value of 3.9 × 10^–4^/ppb for daily 1-hr maximum O_3_ from Ito et al. (2005) for comparison. Our adjoint cost function for the United States includes only O_3_ because no commonly accepted association between NO_2_ and short-term mortality is available for the United States.

Mortality valuation estimates driven by Equation 2 are a function of population demographics. For Canada, we used 2007 total population and annual nonaccidental baseline mortality rates (with no distinction by age category) for each of Canada’s census divisions from AQBAT. For the United States, total population and baseline mortality rates were obtained for each county from the Centers for Disease Control and Prevention (CDC). Nonaccidental mortality rates were calculated from *International Classification of Disease* (ICD)-10 (World Health Organization 1994) codes A–R as in Bell et al. (2004). We applied VSLs in 2011 equivalents (adjusted using the Consumer Price Index) of $5.7M CAD in Canada (from AQBAT) and $8.1M USD in the United States [U.S. Environmental Protection Agency (EPA) 2010]. When influences on the two countries were compared or added, exchange rate parity was assumed.

Through monetary valuation of mortality, we aimed to establish a benefit–cost assessment framework for streamlined comparison between societal benefits and associated pollution abatement costs. We refer to our mortality count valuation as “health benefits” hereafter for simplicity, while recognizing that our calculated values represent a societal willingness to pay to reduce the risk of premature death. Our health benefit estimations are overall conservative in that we are accounting for short-term mortality from gas-phase pollutants without including morbidity or long-term effects. Note that we refer to health benefit influences of marginal source emission reductions when using the term “source attribution.”

*Health benefit estimation case study*. We used the U.S. EPA’s Community Multiscale Air Quality (CMAQ) model (Byun and Schere 2006) and its adjoint for health benefit source attribution. The description and validation of the adjoint of CMAQ has been reported previously (Hakami et al. 2007). The current adjoint model for CMAQ only includes gas-phase processes (chemistry and transport) of 72 active species. Our application of CMAQ was driven by meteorology from the Weather Research and Forecasting (WRF) model (Skamarock et al. 2005) and emissions calculated on a day-by-day, hour-by-hour basis using the Sparse Matrix Operator Kernel Emissions (SMOKE) model (University of North Carolina Institute for the Environment 2009). Emissions were projected to our simulation year from the 2005 National Emissions Inventory (NEI) for the United States and the 2006 National Pollutant Release Inventory (NPRI) for Canada. Our simulation was conducted over a continental domain with a horizontal grid resolution of 36 km (i.e., a matrix of 36 × 36 km grid cells), 34 vertical layers extending into the stratosphere, and for the summer of 2007. When compared to O_3_ observations, our simulations showed a 16.5% mean fractional error (MFE) and +5.5% mean fractional bias (MFB) [see Supplemental Material, p. 3 (http://dx.doi.org/10.1289/ehp.1205561)]. Therefore, our exposure metrics are fairly accurate but slightly overestimated; however, this bias in concentrations is not expected to have a significant impact on source attribution results. Without capturing source influences on exposure to PM (the adjoint of CMAQ for PM is still in development) or other short/long-term effects, we regard our study as a proof-of-concept analysis.

## Results

**Figure 1:**
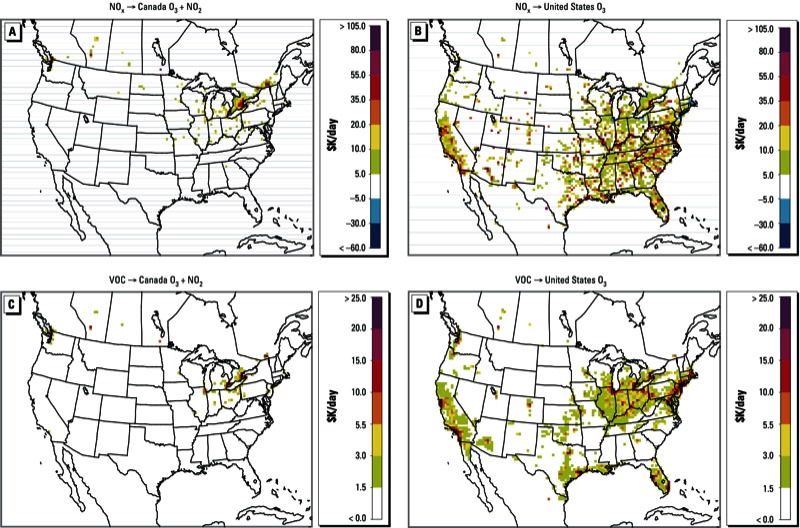
Average daily health bene­fit influences of emissions from individual source locations for Canada (*A,C*) and the United States (*B,D*) estimated for a 10% reduction in anthropogenic emissions of NO_x_ (*A,B*) and VOCs (*C,D*). Health bene­fit influences on Canada account for both O_3_- and NO_2_-related mortality (*A,C)*, whereas influences on the United States account for mortality associated with O_3_ exposure only (*B,D*). Health benefits are average daily influences from 1 July to 30 September 2007. Note that benefits are shown according to the locations of the emissions sources that determine them, rather than the locations that experience the health benefits. For example, influences of both U.S. and Canadian NO_x_ sources shown in *A* indicate nation­wide bene­fits experienced by Canadians only, whereas influences of U.S. and Canadian NO_x_ sources shown in *B* indicate nation­wide benefits experienced by Americans only.

We estimated Canadian and U.S. health benefits from NO_x_ and VOC emission reductions in each location or grid cell (i.e., each 36 × 36 km box) (Figure 1). Estimated health benefit influences are reported in $1,000s per day ($K/day) for a 10% change in emissions in all layers, and they represent daily contributions to annual health benefits (i.e., baseline mortality is scaled to a daily rate). For example, in Figure 1A, a value of $100,000/day in a grid cell indicates that a 10% reduction in NO_x_ emissions from that cell would benefit Canada by $100,000/day in reduced mortality nationwide, whereas in Figure 1B, a value of $100,000/day in a grid cell indicates that a 10% reduction in NO_x_ emissions from that cell would benefit the United States by $100,000/day in reduced mortality nationwide. Note that the adjoint method provides source-specific information but lacks receptor specificity, and therefore, the distribution of benefits across receptors (i.e., mortality reductions according to geographic location) cannot be seen in these results. However, the model does provide a means to quantify national-level benefits resulting from both domestic emission reductions and reductions in emissions in the adjacent country. Health benefits are average daily influences over 1 July 2007 to 30 September 2007 and are reported in each country’s respective currency.

*Attribution of Canadian health benefits to North American sources*. Canadian health benefits from changes in exposure to both O_3_ (daily 1-hr maximum) and NO_2_ as a consequence of reductions in NO_x_ emissions are shown in Figure 1A. Most notable is the tendency of influences to exist in proximity to population centers in Canada, suggesting a strong local component to these health benefits. Although emissions from sources in high-population urban areas will have a greater likelihood of influencing population exposure to O_3_ and NO_2_, their influence can be extended across the nations. Long-range influences of sources from locations in the United States on benefits accrued in Canada reflect the relatively long atmospheric lifetime of O_3,_ whereas influences on NO_2_ occur more locally [for benefits related to O_3_ and NO_2_ separately, see Supplemental Material, Figure S1 (http://dx.doi.org/10.1289/ehp.1205561)]. The largest overall influence came from emissions in Hamilton (upwind of Toronto, Ontario, Canada), reaching $253,000/day (Figure 1A). Significant influences were also seen from emissions in the Quebec City–Windsor Corridor and emissions from the northeastern United States (e.g., $211,000/day in Montreal, Quebec, Canada and $47,000/day in Detroit, MI, USA). VOC emissions had significantly lower estimated influences on mortality (Figure 1C), with the largest benefit seen for emission reductions upwind of the Greater Toronto Area ($54,000/day for a 10% reduction) where a VOC-limited chemical regime exists on many summer days (i.e., production of O_3_ is more affected by VOC availability, rather than NO_x_ concentrations). Canada-wide health benefits have consistently positive sensitivities to anthropogenic VOC emissions across the domain.

*Attribution of U.S. health benefits to North American sources*. Health benefit influences on the United States from anthropogenic NO_x_ emissions are calculated for O_3_ exposure only (based on a 24-hr average metric; Figure 1B). In comparison to results for Canada, contributions of North American NO_x_ emissions toward U.S. mortality valuations are traced to sources dispersed over a wider geographic area and have generally higher magnitudes due to both the larger populations and the higher emissions in the United States. The largest estimated benefits were from reductions in emissions from sources near Atlanta, Georgia ($181,000/day for a 10% reduction in NO_x_ emissions); comparable to the influence seen from NO_x_ emission reductions in Montreal in Figure 1A. We also estimated substantial negative sensitivities or disbenefits from emissions originating in large U.S. cities (e.g., New York, NY, and Los Angeles, CA, at –$681,000/day and –$244,000/day, respectively). These negative influences coincided with NO_x_-inhibited atmospheric conditions where O_3_ production increases as NO_x_ availability decreases; thus reducing NO_x_ emissions-increased O_3_-related mortality. This is in contrast with consistently positive benefits estimated for Canada (Figure 1A), where any disbenefits in O_3_-related mortality under NO_x_-inhibited conditions were offset by concomitant benefits in NO_2_-related mortality. Our estimated benefits for the United States (Figure 1B) do not account for NO_2_ exposure, and thus negative values persisted under NO_x_-inhibited conditions. We also observed that estimated benefits from reductions in VOC emissions (Figure 1D) were significantly higher in magnitude than for Canada, particularly for VOC-limited (or NO_x_-inhibited) metropolitan regions (the largest influences are in New York and Los Angeles, at $294,000/day and $272,000/day for 10% reductions in VOC emissions in each city, respectively).

**Figure 2:**
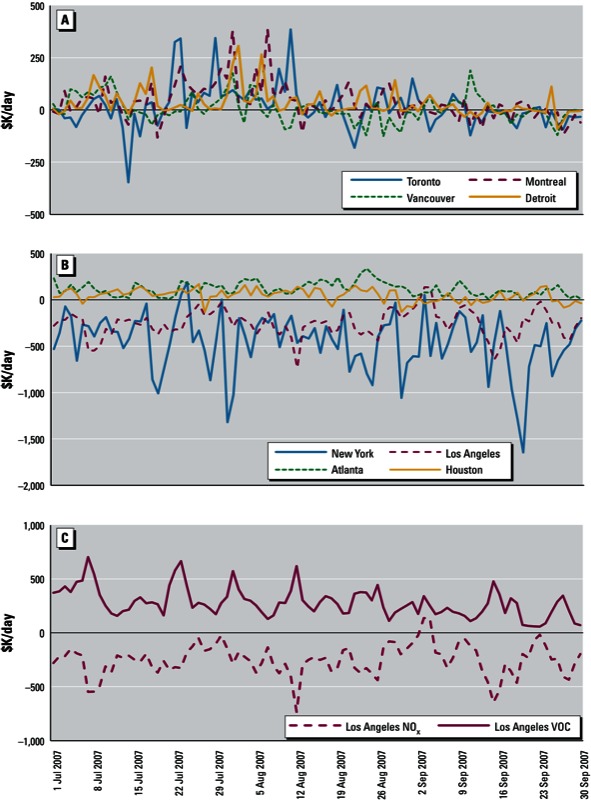
Daily variability of influences from a 10% reduction in anthropogenic emissions of NO_x_ originating from major cities on short-term mortality due to O_3_ exposure in Canada (and Detroit, MI, USA) (*A*) and the United States (*B*) in 2007. (*C*) Daily variability in NO_x_ and VOC influences from Los Angeles on mortality in the United States. Influences are shown for single grid cells coinciding with the center of each city.

A few points about disbenefits from NO_x_ emission reductions in large U.S. cities (Figure 2A) are worth mentioning. First, only O_3_-related mortality was included in our health benefit estimates. If PM-related health effects were considered as well, these disbenefits would be expected to diminish because of reduced inorganic PM concentrations. Second, adjoint sensitivities provide a measure of individual source (or location) contributions that, if considered in isolation, should be regarded as local in nature. In reality, emission reductions are likely to be introduced within a larger regional and/or national context which may alter adjoint source influences, and in some cases may turn disbenefits into benefits. Previous forward sensitivity studies have shown that influences of NO_x_ emissions on O_3_ concentrations remain linear up to about a 30% change in domain-wide NO_x_ emissions (Hakami et al. 2004). Consequently, adjoint sensitivity estimates may not be valid over changes in emissions that are large enough to affect the chemical regime of the atmosphere. Therefore, in the presence of widespread and substantial changes in emissions, a multistep analysis of health benefits (i.e., multiple adjoint simulations for gradually altered emission baselines over time) is more appropriate. Estimation of adjoint sensitivities along the emission control trajectory would result in gradually diminishing disbenefits as changes in emissions become substantial enough to shift the predominant chemical regime in cities away from a NO_x_-inhibited environment. Finally, the results shown in Figure 1 do not consider positive transboundary influences (e.g., the benefits of reduced O_3_ exposure in Europe as a result of reducing U.S. NO_x_ emissions).

*Temporal variability in health benefit influences*. The dependence of atmospheric pollutant transformation and transport on meteorological conditions causes a great deal of day-to-day variability in health benefit attributions. Figure 2 depicts time-variant influences of 10% reductions in NO_x_ or VOC emissions from sources in select cities on Canada-wide and U.S.-wide mortality due to O_3_ exposure. Daily snapshots (i.e., the spatial distribution of influences on specific days) are shown in Supplemental Material, Figure S2 (http://dx.doi.org/10.1289/ehp.1205561).

Significant day-to-day fluctuations in health benefit influences were evident for emission sources in all cities. Reductions in emissions from sources in major Canadian cities (e.g., Toronto and Montreal; Figure 2A) resulted in some days with sizably negative influences on O_3_-related mortality in Canada (though increases in O_3_-related mortality were counteracted by decreases in NO_2_-related mortality that are not shown in Figure 2). In the case of the United States, NO_x_ emission reductions in New York and Los Angeles (Figure 2B) contributed, on average, large disbenefits to national O_3_ mortality. On the other hand, reductions in emissions from sources in or near Atlanta showed consistent benefits on daily O_3_-related mortality due to the abundance of biogenic VOCs and a predominantly NO_x_-limited chemical regime (such that O_3_ production is always expected to decrease as NO_x_ emissions are reduced). Furthermore, strongly NO_x_-inhibited urban cores such as Los Angeles exhibited an inversely correlated behavior between NO_x_ and VOC influences on day-to-day mortality because reductions in NO_x_ will promote O_3_ production (and increase O_3_-related mortality) under these conditions, although reductions in VOCs on the same days will decrease O_3_ production and related mortality (Figure 2C).

The significant daily variability observed in health benefit influences has important policy implications. Air quality decisions are understandably made based on the overall or average estimated impact of pollution control options. However, long-term measures taken based on average conditions may be effective on some days and ineffective on others. Significant day-to-day variability in our estimates suggests that targeted short-term measures guided by health benefit influences may complement long-term strategic planning for air quality improvement. Although air quality forecasting efforts have so far been focused on concentration predictions, forecasting health benefit sensitivities for guiding short-term emission modification seems to be the next logical step.

## Discussion

Figure 1 provides basic aggregate influences on Canadian and U.S. health benefits from various anthropogenic sources in North America. We explore and discuss policy consequences of these results in more detail below.

*Cross-border transport of health benefits*. To assess the impact of cross-border transport on national mortality, we summed health benefit influences coming from emission sources within Canada and the United States separately for two scenarios: *a*) Canadian populations as the receptor for O_3_ and NO_2_ exposure, and *b*) U.S populations as the receptor for O_3_ exposure. These summations should be regarded as marginal influences due to a modest decrease in emissions (i.e., 10%) rather than total contributions (or apportionment) resulting from setting all emissions to zero and thus removing the total influence of each country. As before, we used VSL and epidemiological statistics consistent with the approaches taken and/or time-series studies done in each country.

For Canadian O_3_ and NO_2_ exposure, almost all of the long-range influences from U.S. emissions are due to O_3_. If all NO_x_ sources in the United States reduced emissions by 10%, Canada would experience an average estimated benefit of $3.8M/day (< 1 death/day at a VSL of $5.7M). Similarly, a 10% reduction in all Canadian NO_x_ emissions would produce an average benefit of $4.0M/day on Canadian health. For the exposure of the U.S. population to O_3_, cross-border transport of NO_x_ resulting from a 10% reduction in emissions from Canadian sources would result in an average benefit to the United States of $1.7M/day, whereas the total influence of a 10% reduction in U.S. emissions on American health benefits is estimated to be $51.5M/day (~ 6 deaths/day at a VSL of $8.1M). In comparison with NO_x_, cross-border influences of VOC emissions on both Canadian and U.S. populations are substantially smaller and more local in nature.

The absolute magnitudes of cross-border mortality influences are comparable for the United States and Canada. However, even in the case of Canadian health benefits, there is a significant domestic component. On specific days, cross-border transport of U.S. emissions may have a greater influence on Canadian mortality than domestic emissions [see examples in Supplemental Material, Figure S2 (http://dx.doi.org/10.1289/ehp.1205561)], but in general, we estimate that significant benefits would be gained from domestic emission controls in Canada. Also, an examination of influences by emission release layers shows that surface emissions (layer 1) are by far more influential than elevated sources (layers 2–8) (see Supplemental Material, Figure S3). This suggests that transportation emissions may be more influential on O_3_ (and NO_2_) mortality than industrial sources.

*Effect of averaging period on health benefit influences*. In the results discussed so far here, we have used daily 1-hr maximum O_3_ exposure metrics to estimate benefits for Canada, and 24-hr average O_3_ exposure metrics to estimate benefits for the United States, as these are the common metrics used in each country. Daily average and 1-hr maximum O_3_ concentrations are often correlated, but would respond differently to emission reductions of O_3_ precursors. To explore the impact of the choice of metric on health benefit estimates, we repeat our adjoint calculations for U.S. mortality based on a daily 1-hr maximum O_3_ concentration response factor from Ito et al. (2005). Because Ito et al. (2005) and Bell et al. (2004) used different underlying data, our comparison should be regarded as qualitative; we mainly aim to examine differences in patterns and tendencies.

**Figure 3:**
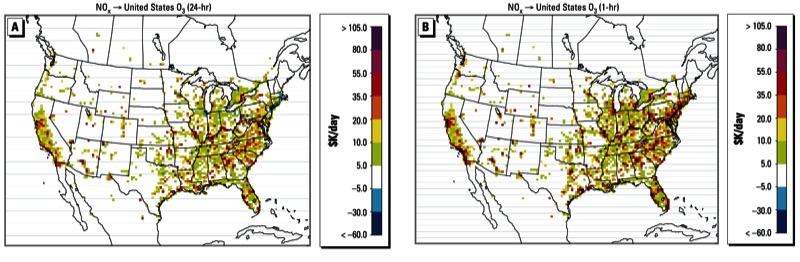
Average daily influences on U.S. short-term mortality estimated for various averaging periods from a 10% reduction in anthropogenic emissions of NO_x_. Health benefit influences are calculated based on 24-hr average O_3_ concentrations (*A*, as in Figure 1B) and daily 1-hr maximum O_3_ concentrations (*B*).

Health benefit estimates based on the 1-hr exposure metric (Figure 3B) are consistently higher than estimates based on the 24-hr average metric (Figure 3A). More important, some locations that exhibit negative sensitivities (i.e., where emission reductions are associated with increased mortality) with the 24-hr averaging period (e.g., around the Great Lakes) have sizable estimated benefits based on a 1-hr metric. This is expected because the daily exposure metric includes nighttime influences when NO_x_ reductions are likely to result in increased O_3_ concentrations, resulting in negative influences on mortality. In contrast, NO_x_ reductions during the day are more likely to have beneficial influences due to reductions in O_3_, except in urban environments that are extremely NO_x_-inhibited. In extremely NO_x_-rich urban cores such as New York or Los Angeles, NO_x_ disbenefits persist (or can become more significant) even with exposure metrics based on 1-hr maximum concentrations. Although we examined only 1-hr and 24-hr metrics for the United States, these diurnal tendencies are an important consideration for the 8-hr O_3_ metric used in regulations.

**Figure 4:**
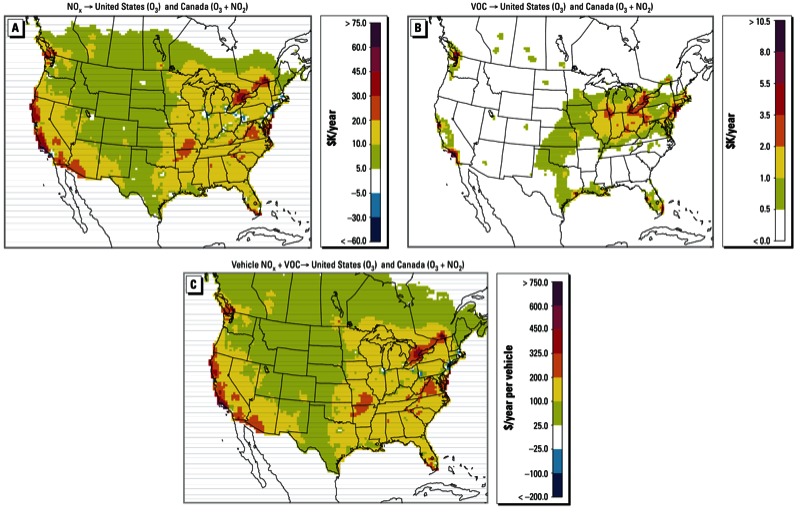
Average yearly influences of 1 metric ton/year reductions in anthropogenic surface-layer emissions of NO_x_ (*A*) and VOCs (*B*) on North American short-term mortality. Unit reductions in emissions are distributed throughout all days and hours of the year based on domain-wide diurnal emission patterns assigned to each grid cell. (*C*) Estimated yearly benefits attributed to elimination of one average vehicle in a given location for both the United States (O_3_) and Canada (O_3_ and NO_2_) combined.

*Health benefit influences of unit source reductions*. Day-to-day and temporal average health benefit influences are a function of *a*) population demographics, *b*) physical and chemical environmental processes that define source-receptor relationships, and *c*) the magnitude of emissions at each source location. In general, emissions of NO_x_ or VOCs from a grid cell will have a relatively large influence if they are large in magnitude, or if there is a large population exposed to their emissions, or both. A grid cell near a populous area has a large potential for influencing human health, but if that location has very little actual emissions, it will exert no influence on estimated benefits. To remove the inherent dependency of sensitivities on the spatial and temporal distribution of emission quantities, we estimated health benefit influences for hypothetical unit reductions in anthropogenic NO_x_ and VOC emissions of 1 metric ton/year (Figure 4A,B). Unit reductions of NO_x_ and VOC emissions are spread evenly throughout all days of the year and are based on domain-wide diurnal emission patterns assigned to all grid cells equally. The resulting influences represent marginal, annual benefits (extrapolated from summer months to the full year) from unit emission reductions at each location, which can be invaluable decision-making parameters. The results depict the overall influence of the same reduction in each source on both Canada (O_3_ and NO_2_) and the United States (O_3_).

These estimated health benefit influences have significantly greater spatial coverage than the estimates shown in Figure 1. Health benefit influences mainly reflect benefits from NO_x_ reductions, and are highest along the Quebec City–Windsor Corridor (Canada) and in California (USA), consistent with the fact that adjoint forcing is driven by downwind populations (largest values of approximately $75,000/year in Santa Barbara, Simi Valley, and west of Montreal, Dorval). As expected, large cities have lower attributed benefits (due to a VOC-limited chemical regime resulting in increased O_3_ concentrations and O_3_-related mortality with reductions in NO_x_). Estimates in Canada are generally larger because they include influences on both O_3_ and NO_2_ exposure.

Using fleet-average emission rates, values in Figure 4A and B can be translated into a benefit attribution map for personal vehicle use (Figure 4C). Values in Figure 4C can be interpreted as the yearly benefit of removing one average vehicle from the road in each grid cell, due to elimination of the vehicle’s NO_x_ and VOC emissions. Yearly benefits are calculated using annual per-vehicle emission rates of 0.010 metric ton/year for NO_x_ and 0.014 metric ton/year for VOCs as averages taken from the mobile emission inventory developed by SMOKE. Some major urban areas in the United States show small estimated influences from transportation (e.g., Los Angeles $0/year), with significant disbenefits estimated for New York (–$750/year), Boston (–$150/year), and a few other cities. In Canada, because of the inclusion of NO_2_ in the adjoint cost function, no disbenefits are observed and estimated urban benefits are substantial, with the largest Canadian influences in Montreal ($770/year), Mississauga, Ontario, Canada ($440/year), and Vancouver, British Columbia, Canada, ($450/year). In the United States, influences from the Pacific Ocean Highway in regions other than Los Angeles and the Bay Area are substantial, ranging between $300/year and $830/year.

## Conclusions

We used the adjoint of CMAQ to estimate nationwide health benefits from reduced O_3_- and NO_2_-related short-term mortality resulting from NO_x_ and VOC emission reductions in each source location. Our modeling period represents a single O_3_ season in 2007, and does not capture inter-annual variability in health benefit influences. Furthermore, while our calculations based on summer months are likely to overestimate annual benefits when extrapolated to the full year, we believe that, overall, we underestimate health benefits in not accounting for morbidity and long-term or PM-related mortality.

Our estimates are affected by various uncertainties in epidemiological values, mortality valuation, emissions characterization, and atmospheric modeling (e.g., representation of complex atmospheric chemistry). Emission uncertainties are of particular importance because they are thought to be the major source of uncertainty in simulated concentrations (Russell and Dennis 2000). Sharp spatial gradients of health benefit influences can only be captured with higher resolution simulations (i.e., a smaller grid cell size). Although these results provide insight into the general behavior of health benefit attributions, they should be regarded as a proof-of-concept demonstration of the adjoint method’s capability to delineate health benefit influences. More conclusive quantification of influences requires further research with high-resolution, multiyear, multipollutant simulations that span all possible health outcomes with adequate consideration for uncertainties.

Our results indicate important tendencies of health benefit influences:

From the day-to-day variability in health benefit influences, we infer that the efficacy of long-term pollution reduction measures could vary greatly in the short term.
We note a sizeable influence of cross-border transport, with the estimated influence of U.S. emissions on Canada being larger than the estimated influence of Canadian emissions on the United States, but comparable in magnitude to the influence of domestic Canadian emissions on Canadian health. From a Canadian perspective, although the tendency to blame poor air quality on emissions in the United States seems somewhat justified, there is significant benefit to be gained from domestic emission controls.
Our results point to substantial differences in the response of exposure metrics to control of emissions when calculated for various averaging periods. These differences could have important regulatory implications and as such, this topic requires further investigation (with inclusion of the 8-hr metric) based on consistent underlying epidemiologic models.
Our estimates suggest that ground-level sources have the largest influences except where significant industrial activity exists. As such, we anticipate a potentially important application of this approach in transportation planning. For example, based on our results, we estimate health benefits of the subway system in Toronto to be approximately $130M/year from reduced short-term O_3_ and NO_2_-related mortality only.
Most important, our results suggest that potential health benefits are substantial and possibly underrepresented in the current benefit–cost analysis frameworks that lack source specificity. For example, the U.S. market-based permit price (the average marginal abatement cost) available to power plants for 1 metric ton of NO_x_ emissions reduction during the O_3_ season in 2007 was approximately $900 (U.S. EPA 2008). By contrast, our corresponding estimated seventh layer (typical effective height for a power plant plume release) health benefit influence for the Ohio River Valley is approximately $11,000/year. Such disparity between marginal abatement costs and marginal benefits can be best addressed using the source-specificity offered by the adjoint approach.

## Supplemental Material

(901 KB) PDF
