# Supplementary material for: Source Attribution of Health Benefits from Air Pollution Abatement in Canada and the United States: An Adjoint Sensitivity Analysis
Source: Environ Health Perspect. 2013 Feb 22;121(5):572–9. doi: 10.1289/ehp.1205561 (PMC3673189; doi:10.1289/ehp.1205561)
Supplement: (901 KB) PDF [file ehp.1205561.s001.pdf]

Environmental Health Perspectives doi:10.1289/ehp.1205561

Supplemental Material

Source Attribution of Health Benefits from Air Pollution Abatement in Canada and the United States: An Adjoint Sensitivity Analysis

Amanda J. Pappin and Amir Hakami

Department of Civil and Environmental Engineering, Carleton University, Ottawa, Canada

Table of Contents

S1. Model Evaluation 3

S2. Supplemental Figures 4

Supplemental Material, Figure S1: Average daily influences on Canada short-term mortality related to (A) O_3 and (B) NO_2 from a 10% reduction in anthropogenic emissions of NO_x........ 4

Supplemental Material, Figure S2: Daily variability of influences from a 10% reduction in anthropogenic emissions of NO_x originating from individual source locations on Canada short-term mortality related to both O_3 and NO_2 exposure. 5

Supplemental Material, Figure S3: Average daily influences of 10% reduction in anthropogenic emissions of NO_x (A,B) and VOCs (C,D) on Canada short-term mortality from surface-level emissions (left) and elevated sources (right). 6

References 7

S1. Model Evaluation

To evaluate the performance of the Community Multiscale Air Quality (CMAQ) model, we compare observation-based O_3 concentrations with model-predicted O_3 concentrations over the July-September 2007 simulation period. Observations of O_3 concentrations are taken from the U.S. EPA's Air Quality System (AQS) (U.S. EPA 2012).

Mean fractional error (MFE) is based on the absolute value of the difference between any pair of model-predicted and observation-based O_3 concentrations (Russell and Dennis 2000):

$$MFE = \frac{2}{N} \cdot \sum_{1}^{N} \frac{|C_m - C_o|}{(C_m + C_o)}$$

where C_m is the model-predicted ground-level O_3 concentration (layer 1) in a particular location and time, C_o is the O_3 concentration observed at a monitor located in the same grid cell and at the same time, and N is the number of observations across all locations and times. If more than one monitor exists at a grid cell, an average of their measurements is used.

Mean fractional bias (MFB) quantifies the directional (i.e., positive or negative) tendency of CMAQ to over or under-estimate O_3 concentrations compared to observations (Russell and Dennis 2000):

$$MFB = \frac{2}{N} \cdot \sum_{1}^{N} \frac{(C_m - C_o)}{(C_m + C_o)}$$

S2. Supplemental Figures

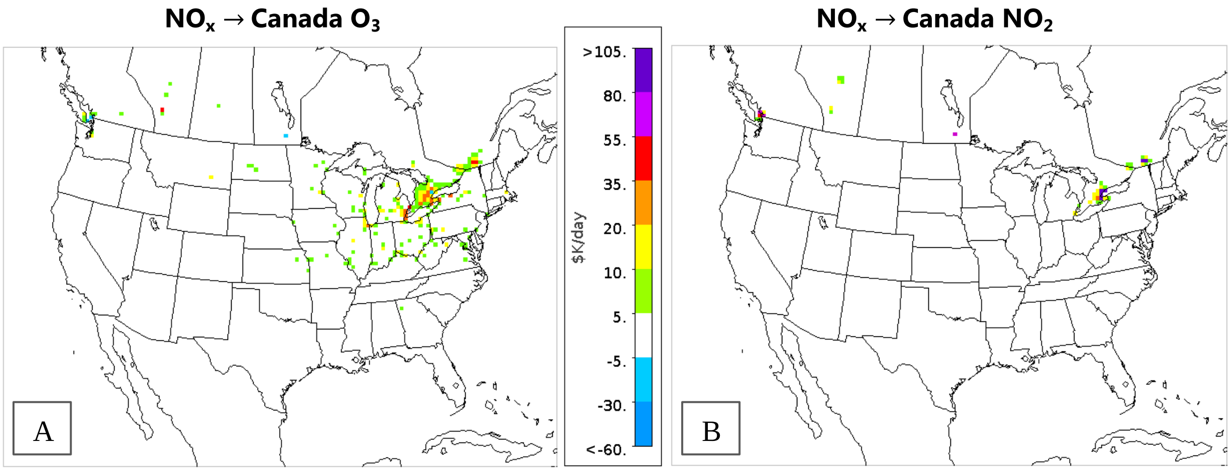

Supplemental Material, Figure S1: Average daily influences on Canada short-term mortality related to (A) O_3 and (B) NO_2 from a 10% reduction in anthropogenic emissions of NO_x. Health benefit influences are estimated based on (A) daily 1-hr maximum O_3 concentrations and (B) 24-hr average NO_2 concentrations separately.

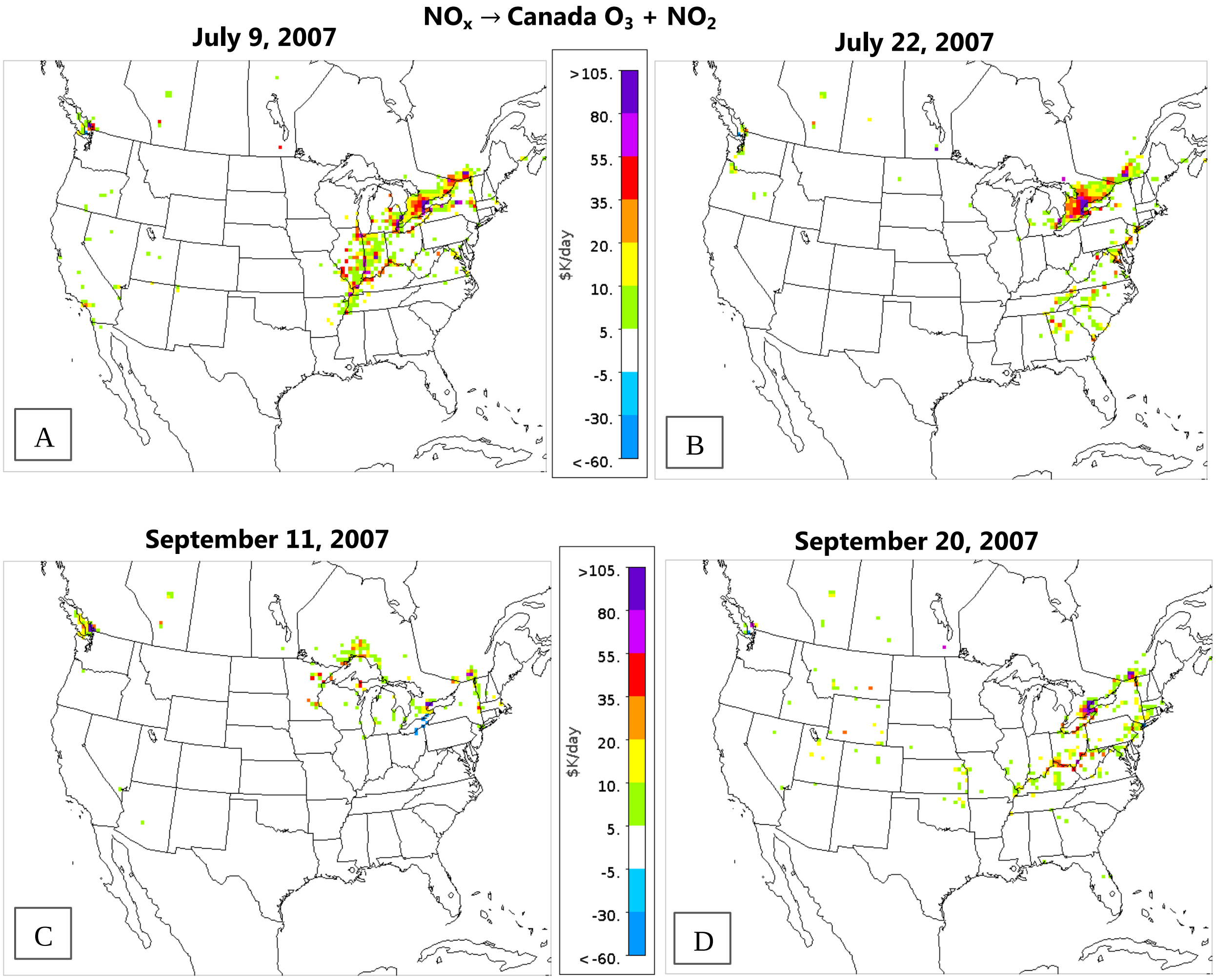

Supplemental Material, Figure S2: Daily variability of influences from a 10% reduction in anthropogenic emissions of NO_x originating from individual source locations on Canada short-term mortality related to both O_3 and NO_2 exposure. Snapshots are shown for (A) July 9, (B) July 22, (C) September 11 and (D) September 20 during 2007.

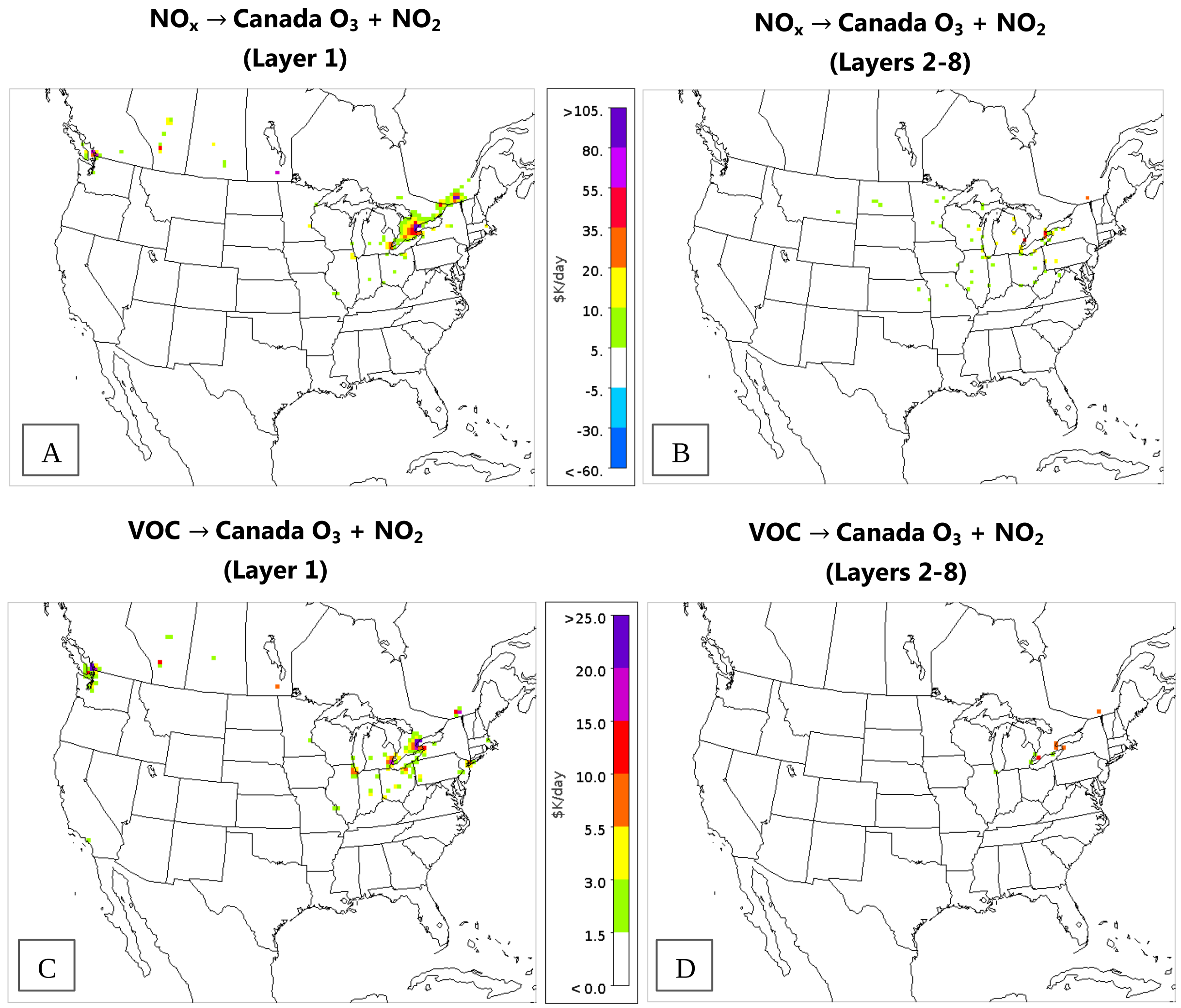

Supplemental Material, Figure S3: Average daily influences of a 10% reduction in anthropogenic emissions of NO_x (A,B) and VOCs (C,D) on Canada short-term mortality from surface-level emissions (left) and elevated sources (right).

References

Russell A, Dennis R. 2000. NARSTO critical review of photochemical models and modeling. Atmos Environ 34:2283-2324.

U.S. EPA. 2012. Technology Transfer Network (TTN) Air Quality System (AQS) Homepage. Available: http://www.epa.gov/ttn/airs/airsaqs/index.htm [accessed 14 February 2013].
